# The Diagnostic Usefulness of ^131^I-SPECT/CT at Both Radioiodine Ablation and during Long-Term Follow-Up in Patients Thyroidectomized for Differentiated Thyroid Carcinoma: Analysis of Tissue Risk Factors Ascertained at Surgery and Correlated with Metastasis Appearance

**DOI:** 10.3390/diagnostics11081504

**Published:** 2021-08-20

**Authors:** Angela Spanu, Susanna Nuvoli, Andrea Marongiu, Ilaria Gelo, Luciana Mele, Andrea De Vito, Maria Rondini, Giuseppe Madeddu

**Affiliations:** 1 Unit of Nuclear Medicine, Department of Medical, Surgical and Experimental Sciences, University of Sassari, 07100 Sassari, Italy; susannanuvoli@gmail.com (S.N.); m.and@live.it (A.M.); ilariagelo@yahoo.it (I.G.); luciana.mele@gmail.com (L.M.); maria.rondini01@ateneopv.it (M.R.); giuseppe.madeddu@email.it (G.M.); 2 Unit of Infectious Diseases, Department of Medical, Surgical and Experimental Sciences, University of Sassari, 07100 Sassari, Italy; andreadevitoaho@gmail.com

**Keywords:** ^131^I-SPECT/CT, ^131^I-WBS, differentiated thyroid cancer, thyroglobulin, tissue risk factors

## Abstract

^131^I Single-photon emission computerized tomography/computerized tomography (SPECT/CT) in the management of patients thyroidectomized for differentiated thyroid carcinoma (DTC) was further investigated. Retrospectively, 106 consecutive DTC patients were enrolled at the first radioiodine ablation, 24 at high risk (H), 61 at low risk (L) and 21 at very low risk (VL). ^131^I whole-body scan (WBS) and SPECT/CT were performed after therapeutic doses using a hybrid dual-head gamma camera. At ablation, SPECT/CT correctly classified 49 metastases in 17/106 patients with a significantly (*p* < 0.001) more elevated number than WBS which evidenced 32/49 foci in 13/17 cases. In this case, 86/106 patients could be monitored in the follow-up including 13/17 cases with metastases already at post-therapeutic scans. SPECT/CT after radioiodine diagnostic doses more correctly than WBS ascertained disease progression in 4/13 patients, stable disease in other 4/13 cases and disease improvement in the remaining 5/13 cases. Further 13/86 patients with only residues at post-therapeutic scans showed at SPECT/CT 16 neck lymph node (LN) metastases, three unclear and 13 occult at WBS. Significant involvement of some tissue risk factors with metastasis appearance was observed, such as minimal extrathyroid tumor extension and neck LN metastases. These risk factors should be carefully considered in DTC patient follow-up where ^131^I-SPECT/CT routinely use is suggested as a support tool of WBS.

## 1. Introduction

Thyroid cancer represents the most common endocrine tumor and its incidence continues to rise; based on histology, differentiated thyroid carcinomas (DTC) comprise the majority of thyroid cancers (about 90%) and include more frequently papillary, follicular and Hürthle cell carcinomas [1,2,3,4]. These types of carcinomas generally have a good prognosis, but the presence of neck lymph node (LN) metastases can be correlated with tumor recurrences [5,6,7,8,9,10] and distant metastases can cause a significant disease progression [3,11,12]. Moreover, extrathyroid tumor extension (ETE) has been recognized as a prognostic factor of DTC [13,14]. However, while extended ETE (eETE) proved a significant worsening of the prognosis [13,14,15,16], the impact of minimal ETE (mETE) is controversial presenting an increased risk of recurrence in some studies [8,17,18,19,20,21] and vice versa reporting no significant impact on disease-free survival in other studies [13,22,23,24,25,26,27,28]. DTC has slow growth and recurrences and metastases can appear and become clinically evident even many years later; thus, the carcinomas can require a long-term follow-up to guarantee careful surveillance of the affected patients [10,29]. Generally, the patients undergo total thyroidectomy followed by ablation with ^131^I-therapeutic dose; however, in patients with papillary microcarcinoma without metastases, only a partial thyroidectomy can be performed with the absence of ablation and with a careful follow-up [30]. ^131^I whole-body scanning (WBS) has been considered for many years the routine diagnostic procedure to identify thyroid tissue residues and both local and distant metastases after thyroidectomy [31,32,33], the procedure being performed in association with the serum thyroglobulin serum assay and radiologic procedures. The higher performance of WBS is achieved even more using a radioiodine therapeutic dose giving important information on patient staging [34]. However, some limitations can be attributed to planar WBS: low sensitivity and difficulty both to specify anatomic localization of radioiodine-avid foci and to characterize the lesions making image interpretation more difficult. Planar WBS often can also not distinguish physiologic radioiodine-avid foci from metastatic lesions leading to misinterpretation and false-positive results, in particular in the neck and in the thorax where the differentiation between physiologic tissues and metastases are even more difficult [35,36,37]. More recently, ^131^I-SPECT/CT has dramatically improved WBS image interpretation; the procedure presents a better contrast resolution than planar acquisition also obtaining functional and cross-sectional anatomic fusion images. Thus, SPECT/CT proved capable of improving the performance of planar WBS in detecting radioiodine-avid foci, increasing sensitivity and accuracy and allowing precise anatomic localization and characterization of the lesions thus improving DTC staging and subsequent patient management. The better performance of SPECT/CT in respect of planar WBS has been ascertained either in pre [3,38,39] and post [40,41,42,43,44,45] radioiodine ablation as well as during long-term follow-up [9,10,46,47,48,49,50].

^124^I-PET/CT has also been used to detect metastases from DTC offering superior imaging characteristics in respect of ^131^I-scans with higher sensitivity and spatial resolution, but with higher cost in respect of ^131^I iodine [51]. However, ^124^I has a complex decay schema, which creates challenges in optimizing imaging. In addition, for a wide use of ^124^I-PET/CT, it is necessary a more commercial availability of the radiopharmaceutical besides creating an imaging protocol which has not yet been established. Finally, it is hoped that the cost of the procedure will be lower than the current ones. 

The present retrospective study aimed to evaluate the incremental diagnostic value of ^131^I-SPECT/CT in respect of WBS after a therapeutic dose of radioiodine during ablation to obtain the most correct disease staging of DTC patients after thyroidectomy and to plan their most appropriate management during the follow-up using in this phase SPECT/CT after radioiodine diagnostic dose. Moreover, it has also evaluated which risk factors identified at surgery seem to be more correlated to the appearance of recurrences and metastases.

## 2. Materials and Methods

### 2.1. Patients

We retrospectively enrolled 106 adult patients, submitted to total thyroidectomy for DTC and undergone the first radioiodine ablation. The characteristics of the patients are reported in Table 1.

In this case, 29 of the patients were males and 77 females; 33/106 (31.1%) of these had an age < 45 years and 73/106 (68.9%) ≥ 45 years. At surgery, in 98 cases, a papillary carcinoma (PC) was ascertained, in three cases a follicular carcinoma (FC) and in five cases a Hürthle cell carcinoma (HCC). In 42/106 (39.6%) patients tumor size was ≤10 mm and in 64/106 (60.4%) >10 mm. In 44/106 (41.5%) patients the carcinoma was multifocal and in 62/106 (58.5%) was unifocal. Moreover, in 21/106 (19.8%) patients, ETE was present, in 20 cases of whom with mETE which, as known, is usually characterized by primary tumor extension to the sternothyroid muscle and/or peri-thyroid soft tissue, and in one case with eETE usually characterized by primary thyroid extension to subcutaneous soft tissue, trachea, larynx, esophagus or laryngeal nerve [52]. In 14/106 (13.2%) cases neck cervical LN metastases were ascertained and in 2/106 (1.9%), distant metastases were present. Five/106 (4.7%) patients were also affected by Graves-Basedow disease (GB) and 20/106 (18.9%) by Hashimoto thyroiditis (HT); of the former five patients, one also had distant metastases and two had mETE. Of the latter 20 cases, 12 also had a multifocal disease and one of these also mETE, while 3 only had mETE and one had both neck LN metastases and mETE. In accord with the classification of the European Thyroid Cancer Taskforce [53], at surgery, 29/106 (27.4%) patients were classified as at high risk (H), 56/106 (52.8%) at low risk (L) and 21/106 (19.8%) at very low risk (VL). The carcinoma was classified as very low risk (unifocal T1 [≤1 cm] N0M0 and there was no extension beyond the thyroid), at low risk (T1 [>1 cm] N0M0, T2N0M0 or multifocal T1N0M0) or at high risk (any T3 or T4 as well as any T with N1 or M1). 

All patients after total thyroidectomy underwent complete thyroid hormone withdrawal for four weeks before radioiodine therapy for endogenous thyroid-stimulating hormone (TSH) stimulation; moreover, the patients had a low iodine diet for two weeks before radioiodine therapy. The patients, in hypothyroidism condition just before oral administration of radioiodine therapeutic dose of 1.85–5.66 GBq, underwent the measurement of urinary iodine excretion (ioduria) and the assay of serum TSH, thyroglobulin and anti-thyroglobulin antibodies (AbTg). TSH levels were always more elevated than the arbitrary levels of 50 µU/mL and those of ioduria were less than 300 µg/L; the cut-off of thyroglobulin was 0.2 ng/mL. High AbTg levels were present in 6/106 patients.

In this case, 86 of the patients could be monitored during a long-term follow-up submitting them to ^131^I-WBS and SPECT/CT after a diagnostic dose of radioiodine (185 MBq).

All patients routinely underwent neck ultrasound that we consider one of the more valuable procedures in the follow up of DTC patients to contribute in identifying neck lymph node metastases, but the data have not included in the text since these are worthwhile-beyond the scope of this study. 

### 2.2. Imaging

All the patients had imaging acquisition 5–7 days after oral administration of therapeutic radioiodine dose. First, ^131^I-WBS was acquired by using a dual-head variable-angle gamma camera (Infinia™ Hawkeye™, General Electric Healthcare, Milwaukee, WI, USA) with high energy, parallel-hole collimators, setting the 131I photon peak (364 Kev) and 20% energy windows. Anterior and posterior images were acquired at a speed of 10 cm/min for a total time of 20 min (matrix: 1024 × 256) with the patient lying in a supine position, using a special vacuum cushion to stabilize the neck position. Immediately after WBS, in the same patient position, SPECT/CT scan from the skull base to the diaphragm was performed interesting neck and chest regions but also other suspected regions under the diaphragm; SPECT was always obtained first followed by a CT scan. SPECT images were acquired around 360° using steps of 3 degrees (40 s/step), a matrix of 128 × 128 and 1–1.2 zoom factor. 

CT scan had an X-ray tube and detector array rotating together in a fixed geometry at 2.0 rpm for a 90° L-mode scan. Multiple CT slices were obtained in helical mode (four 5-mm-thick slices obtained simultaneously, with beam coverage of 2 cm in each gantry rotation and reconstructed online to a 512 × 512 image matrix). The parameters were 140 keV, up to 2.5 mA (total acquisition time 4–5 min). The CT scan was acquired within 4.5 min. Cross-sectional attenuation images (128 × 128 image matrix), in which each pixel represents imaged tissue attenuation, were generated in all cases. SPECT was always acquired first, followed by CT and the SPECT images were reconstructed with the iterative method (ordered-subsets expectation maximization) and fused with the CT images using a dedicated software package (GE Healthcare Xeleris™ Workstation; General Electric Healthcare, Milwaukee, WI, USA). Transversal, coronal and sagittal SPECT and CT images were obtained.

The 86 patients monitored in the follow-up underwent further WBS and SPECT/CT after a diagnostic radioiodine dose of 185 MBq. For these cases, the same modality of patient preparation was used as well as the same characteristics of acquisition and elaboration of WBS and SPECT/CT, which were performed 24–48–72 h after radioiodine ingestion. The only exception resulted in the speed of acquisition of WBS that was 5 cm/min for a total time of 40 min. In this case, 64/86 patients were in hypothyroidism condition after hormone therapy withdrawal, while 22/86 cases received recombinant human TSH stimulation according to standard procedures. 

The study has been performed for clinical purposes according to Helsinki Doctrine (as revised in 2013) and its later amendments or ethical standards. Ethical approval was waived as this was a retrospective study involving data analysis in an anonymous form. All patient data were treated carefully following the Local Privacy Rules and Regulations. 

SPECT/CT together with WBS after radioiodine therapeutic dose at the first ablation or after a diagnostic dose during follow-up is routinely performed in the Unit of Nuclear Medicine, site of the present study, since 2006. 

### 2.3. Image Analyses

^131^I-WBS and SPECT/CT images were analyzed by three nuclear medicine physicians (A.S., S.N. and G.M. with about 15 years of experience in ^131^I-SPECT/CT and more than 30 years in WBS interpretation); they were aware of the reason for the exams but blinded with the results of previous imaging investigations. Inter-observer variability was very low and disagreements were resolved by consensus. The uptakes of ^131^I at WBS higher than the surrounding background were considered normal when distributed in normal tissue or physiologic structures and suggestive for metastases when non-compatible with physiologic storage locations. Neck foci sited in the median region corresponding to thyroid bed or thyroglossal duct were considered remnants and when sited in the nasopharynx, in the superior esophagus and trachea were considered physiologic uptakes; radioiodine uptakes in the nose and both regions of salivary glands also were classified as normal or physiologic. Foci located laterally in the neck and outside the thyroid bed were categorized suggestive for LN metastases and foci with no precise anatomic site or characterization were interpreted as unclear for lesions. Planar WBS foci in the lungs were considered positive for metastases, while when the site was not easily differentiable between mediastinum and lungs the foci were classified unclear. The foci outside the neck, in mediastinum and lungs, were considered suggestive for distant metastases when physiologic uptakes could be excluded. The results of planar WBS were compared with those of SPECT/CT in the thyroid bed, in the neck outside the thyroid bed, in the chest and eventually in other distant sites. SPECT/CT additional value in respect of WBS was defined when it obtained better identification of the foci, precise anatomic localization and characterization, and a correct differentiation between metastatic lesions and normal tissues or physiologic uptake. 

### 2.4. Outcome

Metastasis status evaluated by SPECT/CT results was confirmed by histopathologic findings or by clinical exams, radiologic imaging and serum thyroglobulin assay in the follow-up of 8–60 months when histology was not available.

### 2.5. Statistics

Data were elaborated as percentages on total numbers. McNemar test was used to assess the existence of disagreement between WBS and SPECT/CT data. Categorical variables were evaluated with the Fisher chi-squared test. Univariate logistic regression analysis was conducted to evaluate the variable influence on metastasis development. The statistical significance level was established as *p* < 0.05. The statistical software used was STATA 16.1 (StataCorp LLC, College Station, TX, USA)

## 3. Results

At both procedures SPECT/CT and WBS the radioiodine-avid foci resulted concordantly absent in 5/106 patients with undetectable thyroglobulin levels, excluding tissue residues and malignant lesions. As illustrated in Table 2, SPECT/CT detected 260 foci in 101/106 patients, while WBS identified 231 foci in 97/106 cases (*p* < 0.01).

Both procedures concordantly classified 186 foci such as 172 residues, 8 lung metastases, 5 soft tissue (ST) metastases and one cutaneous contamination in 85/106 patients. SPECT/CT also characterized 42 foci unclear at WBS in 19 patients as 24 residues in the thyroid bed and 10 LN metastases in the neck, the latter being classified as 7 laterocervical (LTC), 1 sub-mandibular (SM), 1 paratracheal (PT), 1 supraclavicular (SC). Six/42 foci were classified as LN metastases outside the neck, such as two LN of the mediastinum (M) and four abdominal-pelvis (AP); moreover, one/42 foci was bone metastasis and one physiologic uptake in the stomach. SPECT/CT also identified 29 foci occult at WBS in 18 patients as 12 residues, 12 LN metastases in the neck (9 LTC, 3 PT) and one LN metastasis outside the neck (AP), and four lung metastases. Furthermore, SPECT/CT changed WBS classification of three further foci (3 W) wrongly considered being one residue in the thyroid bed in a patient, lung metastasis in another patient and bone metastasis in the remaining case. The first focus was changed in neck LN metastasis (SM), the second focus in muscle metastasis in the thorax (TM), while the third focus was evidenced in the vertebral disk of the 6th thoracic spine by SPECT/CT and was considered benign etiology. This focus resulted negative at both CT and at a bone scan and disappeared at a following SPECT/CT with a decline of thyroglobulin levels from 1.2 ng/mL to undetectable values.

In 25/101 patients with radioiodine uptakes at SPECT/CT, the foci were single and in two of these cases, metastases were ascertained (1 M metastasis and one lung metastasis) that resulted unclear and occult, respectively, at WBS. 

As reported in Table 3, globally, SPECT/CT correctly classified 49 malignant foci in 17/106 patients, who were affected by PC, two of whom with follicular variant; 10 patients had an age ≥ 45 years and seven an age < 45 years.

**Figure 1:**
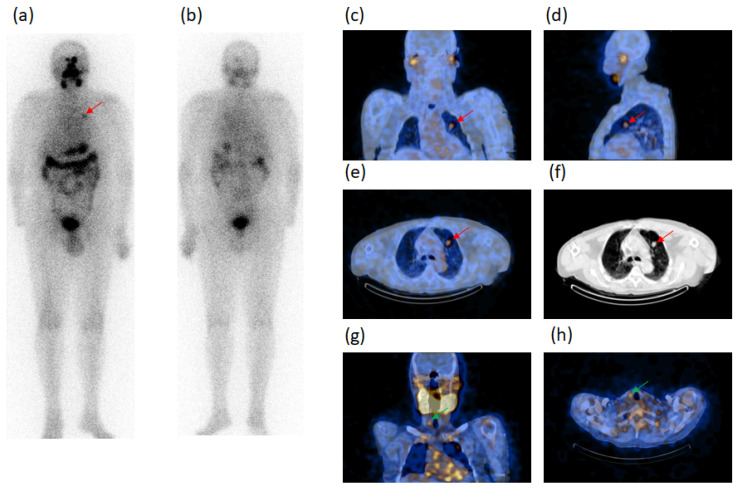
A 73-y-old male patient (case n. 8, Table 3), thyroidectomized for thyroid papillary carcinoma, follicular variant, tumor diameter 25 mm, at the first radioiodine ablation. ^131^I-WBS after a therapeutic dose, in both anterior (**a**) and posterior (**b**) views, showed one radioiodine-avid focus in left hemithorax suspected of metastasis (red arrow). Fused SPECT/CT images in coronal (**c**), sagittal (**d**) and transaxial (**e**) slides confirmed the focus (red arrows) specifying the anatomic site (anterior segment of the superior lobe) and characterizing it as lung metastasis, as also confirmed at CT (**f**). In addition, fused SPECT/CT images in coronal (**g**) and transaxial (**h**) slides evidenced another radioiodine-avid focus (green arrows) in the neck corresponding to a right paratracheal lymph node occult at WBS and classified as metastasis. Thyroglobulin levels in hypothyroidism condition: 1362 ng/mL. AbTg: Absent.

Tumor size was ≤10 mm in six cases and >10 mm in 11 cases. SPECT/CT identified a significant (*p* < 0.001) more elevated number of neoplastic lesions in respect of WBS which evidenced 32/49 malignant foci in 13/17 patients, all positive at SPECT/CT; 13/32 of malignant foci were concordantly evidenced with the two procedures (8 lung metastases and 5 ST metastases). One of these lung foci ascertained in case n. 8, is illustrated in Figure 1.

**Figure 2:**
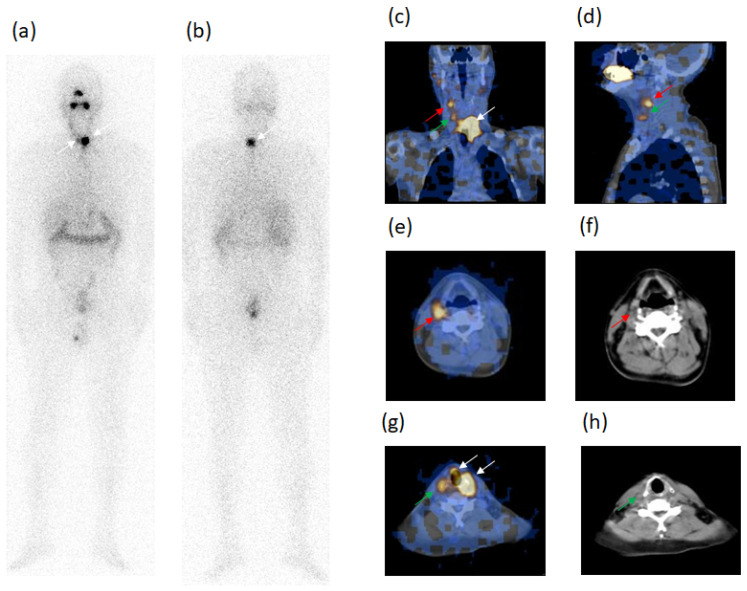
A 42-y-old male patient (case n. 2, Table 3), thyroidectomized for papillary carcinoma, tumor diameter 14mm, at the first radioiodine ablation. ^131^I-WBS after a therapeutic dose, in both anterior (**a**) and posterior (**b**) views, showed two radioiodine-avid foci of different dimension and activity in the median region of the neck (white arrows), better evidenced in anterior view and classified as tissue residues in thyroid bed; moreover, a slightly heterogeneous radioiodine uptake was ascertained in right laterocervical region classified as unclear. Fused SPECT/CT images in coronal (**c**), sagittal (**d**) and transaxial (**e**–**g**) views evidenced the aforementioned two foci (white arrows) in the thyroid bed confirming the classification as residues. SPECT/CT also identified two further circumscribed foci (red and green arrows) in right laterocervical region, whose uptakes were considered unclear by WBS, classifying these as lymph node metastases, as confirmed by CT (**f**–**h**). Thyroglobulin levels in hypothyroidism condition: 29.3 ng/mL. AbTg: absent.

However, WBS classified as unclear 17/32 foci, such as 10 neck LN metastases (7 LTC, 1 SM,1 PT, 1 SC), two of the LTC foci evidenced in case n. 2 are shown in Figure 2 and 7 metastases outside the neck (2 M, 4 AP, 1 bone). These 17 foci were only characterized as metastases by SPECT/CT.

Moreover, WBS wrongly classified 2 malignant foci (2 W), as previously mentioned, and missed 17 further foci corresponding to 13 LN metastases (9 LTC, 3 PT, 1 AP) and 4 lung metastases, only detected by SPECT/CT. At surgery, 6/17 patients with metastases had carcinomas with mETE and 5/6 of these had loco-regional LN metastases. One of the latter five cases had a multifocal disease and one HT, too; 1/17 cases had carcinoma with eETE and neck LN and distant metastases. Of the remaining 10/17 patients, 6 had multifocal PC (one of these also had neck LN metastases, and two were affected by HT) and four had unifocal intra-capsular carcinomas. One of the latter cases also had GB disease and distant metastases and another patient had HT. Thyroglobulin levels before therapeutic WBS and SPECT/CT were ≥10 ng/mL in 10/17 patients, between 2.5 to 5.0 ng/mL in one patient, <2.5 ng/mL in three patients, and undetectable in the remaining three patients (2L, 1 VL). The patient VL had high levels of AbTg. In the 10 patients with metastases occult at WBS, thyroglobulin levels were undetectable in the one case with high AbTg, <2.5 ng/mL in three cases and ≥10 ng/mL in the remaining six cases.

SPECT/CT had an incremental value over WBS in 25.5% of 106 patients and changed the classification and therapeutic management in 16.03% of cases. SPECT/CT changed neck LN and distant metastasis classification performed at the surgery in 11 cases: from N0 to N1 in seven patients and from M0 to M1 in four patients, contributing to modify in these cases therapeutic and management strategy during follow-up.

### Follow-Up

In this case, 86/106 patients could be monitored in the follow-up and were submitted to ^131^I-WBS and SPECT/CT after radioiodine diagnostic dose. The patients also included 13/17 cases in whom metastases had been already ascertained after therapeutic dose for radioiodine ablation and reported in Table 3 (cases 1–13). However, four/17 patients reported in the same Table (cases 14–17) were lost for the follow-up as well as other 16/106 patients without metastasis at post-therapeutic WBS and SPECT/CT at ablation.

In respect of the results of post-therapeutic WBS and SPECT/CT exams, Table 3 also shows the behavior of the 13 patients (cases 1–13) during follow-up. All 13 patients had a PC and two of these were follicular variants (PCFV).

**Figure 3:**
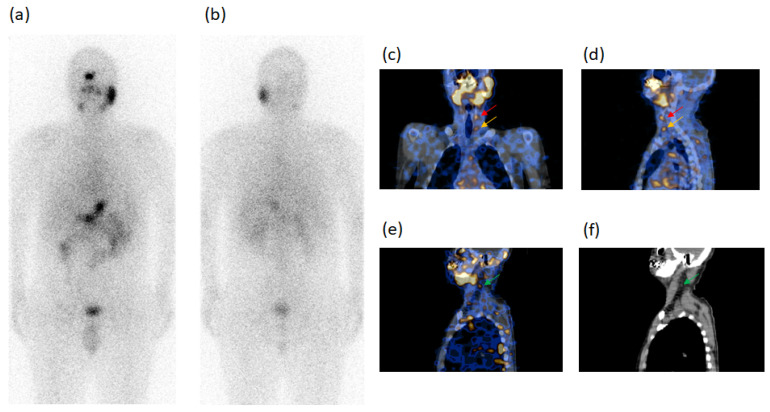
The same patient described in Figure 2 (case n. 2, Table 3) was re-evaluated in the follow-up. WBS in both anterior (**a**) and posterior (**b**) views did not show any radioiodine avid foci to consider as residues or malignant lesions. Fused SPECT/CT images in coronal (**c**) and left (**d**) and right sagittal (**e**) views excluded the two malignant foci evidenced in basal condition. However, it identified three new foci, two of which in the left laterocervical region (red and yellow arrows) and another in right laterocervical region, the latter better evidenced in sagittal right view (green arrow) and confirmed by CT (**f**). These foci were classified as lymph node metastases in disease progression. The patient was treated again with a therapeutic dose of radioiodine. Thyroglobulin levels in hypothyroidism condition: 36 ng/mL. AbTg: absent.

Four/13 patients presented disease progression (cases n. 1–4). In one of the four patients (case n. 1) also affected by HT and previously N1 at radioiodine ablation, the two neck LN ascertained at SPECT/CT at ablation were not more evident. Three new LN metastases were present at follow-up, all of these occult at WBS and with thyroglobulin persistently undetectable but with high AbTg already at surgery and therapeutic scintigraphy. The number of neck LN metastases changed in another of the 4 patients (case n. 2) with the disappearance of 2 metastases ascertained at ablation, and previously shown in Figure 2, and the appearance of three new metastases (occult at WBS) at follow-up, confirming thyroglobulin levels > 10 ng/mL. This case is shown in Figure 3 during follow-up.

A further case of 4 patients (case n. 3) changed classification from N0 to N1 with the appearance of one new LTC metastasis (occult at WBS) and confirming M1 classification with one M metastasis (unclear at WBS), while thyroglobulin value changed from undetectable to 5.0–10 ng/mL. The remaining one/4 patients (case n. 4) changed classification from M0 to M1 with the appearance of one lung and one bone metastasis (both occult at WBS) and confirming N1 classification with 2 persistent LTC metastases to which 1 new SM metastasis (occult at WBS) was added, while thyroglobulin levels persisted >10 ng/mL.

Other 4/13 patients (cases n. 5–8), had a stable disease confirming 1 PT metastasis in one case (case n. 5) at SPECT/CT and occult at WBS with a persistent value of thyroglobulin between 2.5 to 5.0. One PT, occult at WBS, was evidenced at SPECT/CT in another case (case n. 6) with a reduction of thyroglobulin levels from >10 to 5–10 ng/mL. Moreover, distant metastases persisted in a further case (case n. 7), such as one LTC, one bone, five AP, one lung and five AP metastases at both SPECT/CT and WBS, although the latter was unclear or occult in some lesions, with thyroglobulin value persistently >10 ng/mL. One PT metastasis and one lung metastasis in the remaining case (case n. 8) were confirmed with thyroglobulin value always >10 ng/mL.

The remaining 5/13 patients (cases n. 9–13), three of whom also affected by HT (cases n. 9, 10, 11), presented an improvement of clinical conditions and functional imaging in respect of the previous data at post-therapeutic exams. A reduction of the number of neck LN metastases from 3 LTC to 2 LTC in one case (case n. 9) was observed at SPECT/CT, while persistently occult at WBS, with thyroglobulin values reducing from >10 ng/mL to 5–10 ng/mL. Moreover, a reduction from two LTC to one LTC in another case (case n. 10) was ascertained at SPECT/CT, always occult at WBS, with thyroglobulin level decrease from >10 to <2.5 ng/mL. The disappearance of three LTC and the persistence of one PT was evidenced in a further case (case n. 11) at SPECT/CT, but always occult at WBS, with thyroglobulin levels decreasing from <2.5 ng/mL to undetectable. In the case n. 12 also affected by GB, the disappearance of one TM and the reduction from five to one lung metastasis was observed, the latter evidenced at both SPECT/CT and WBS, with thyroglobulin decrease from >10 to 5–10 ng/mL. Moreover, the absence of loco-regional LN metastases changing the previous classification N1 to N0 was observed in the remaining case (case n. 13, VL, T1aN0M0) from 1 SM to absence of metastasis and with reduction of thyroglobulin levels from >10 ng/mL to undetectable levels.

In this case, 13 patients, who had shown only residues at WBS and SPECT/CT after therapeutic radioiodine dose, not changing the TNM and risk stratification ascertained at surgery, underwent metastases during follow-up. As shown in Table 4, at surgery, these 13 patients had papillary carcinomas, six had an age < 45 years and seven an age ≥ 45 years.

In particular, the first three/13 patients had unifocal papillary microcarcinoma without any other tissue alterations and with thyroglobulin levels undetectable in two cases (case n.1 and case n. 2) at radioiodine ablation and undetectable but with high levels of AbTg in the third case (case n. 3); these three patients were VL and T1aN0M0. Another case/13 patient (case n. 4) had unifocal carcinoma with also mETE and GB and with thyroglobulin levels of 2.5–5.0 ng/mL in the therapeutic phase. Further 2/13 patients presented mETE in unifocal carcinoma in one case (case n. 5) and multifocal in the other (case n. 6) with thyroglobulin levels of 2.5–5.0 ng/mL. Other 5/13 patients had unifocal carcinomas, 1/5 with thyroglobulin undetectable (case n. 7) and 2/5 with undetectable thyroglobulin but with high levels of AbTg (case n. 8 and case n. 9, respectively). Another 1/5 cases with thyroglobulin levels <2.5 ng/mL (case n. 10) and the remaining 1/5 cases with also mETE and undetectable thyroglobulin levels (case n. 11) were observed. The remaining 2/13 patients had multifocal carcinomas with thyroglobulin levels undetectable (case n. 12) and between 2.5–5.0 ng/mL (case n. 13). According to risk stratification, four patients were H, 6 L and three VL.

**Figure 4:**
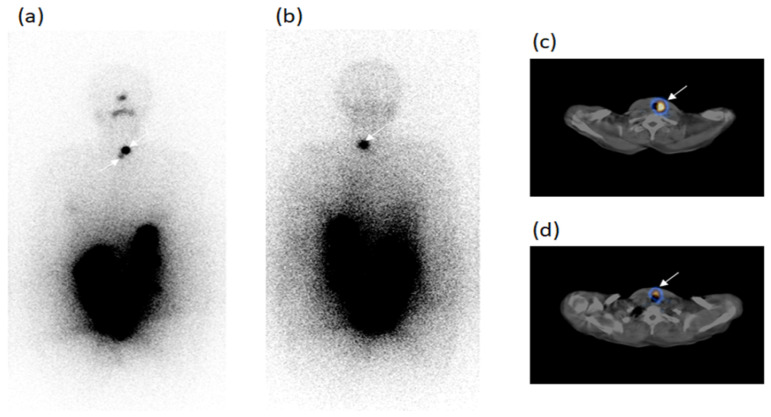
A 60-y-old female patient (case n. 13, Table 4) submitted to total thyroidectomy for multifocal-bilateral papillary carcinoma, tumor diameter 15 mm, at the first radioiodine ablation.^131^I-WBS after therapeutic radioiodine dose, in both anterior (**a**) and posterior (**b**) views evidenced two radioiodine-avid foci of different activity in the median region of the neck classified as tissue residues. Fused SPECT/CT images in transaxial slides (**c**,**d**) also identified the two foci evidenced by WBS confirming residual origin. Both WBS and SPECT/CT excluded the presence of foci doubtful of malignant lesions. Thyroglobulin levels in hypothyroidism condition: 4.2 ng/mL. AbTg: absent.

**Figure 5:**
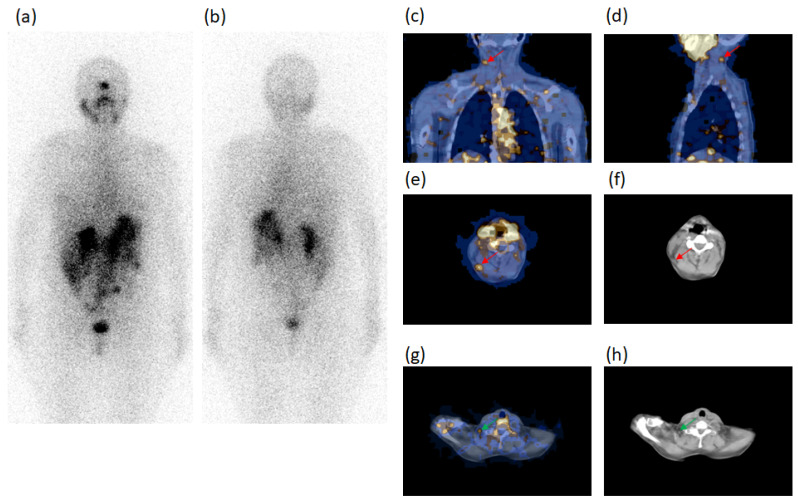
The same female patient described in Figure 4 (case n. 13, Table 4) was also evaluated during follow-up. ^131^I-WBS after diagnostic dose of radioiodine in both anterior (**a**) and posterior (**b**) views did not evidence the residue foci ascertained at the exam performed after therapeutic dose and excluded the presence of other foci doubtful of malignant lesions. Fused SPECT/CT images in coronal (**c**), sagittal (**d**) and transaxial (**e**) slides did not evidence the two foci classified as residues at the previous exam during ablation. However, SPECT/CT identified two new foci, one in right laterocervical region (**e**, red arrow) and the other in right supraclavicular region (**g**, green arrow), both of these occult at WBS. These foci, confirmed at CT (**f**,**h**), were classified as lymph node metastases. The patient was treated with a radioiodine therapeutic dose again. Thyroglobulin levels in hypothyroidism condition: 13 ng/mL. AbTg: absent.

In the follow-up, SPECT/CT after radioiodine diagnostic dose evidenced 16 metastatic foci in the 13 patients, such as eight LTC, seven SM and one SC metastases; three of these foci were unclear and 13 occult at WBS. Six/13 patients (cases n. 1–6) showed 1 SM each at SPECT/CT; the SM was unclear at WBS in case n. 1 and occult in case n. 2 with thyroglobulin levels <2.5 ng/mL in both cases. SM was occult at WBS in case n. 3, with thyroglobulin undetectable but with high values of AbTg, as well as in cases n. 4, n. 5, n. 6 with thyroglobulin levels of 2.5–5.0 ng/mL. Other 4/13 patients had one LTC metastasis each at SPECT/CT, but occult at WBS, in cases n. 7 and n. 9 and unclear in cases n. 8 and n. 10 with thyroglobulin levels <2.5 ng/mL (case n. 7) undetectable but with high AbTg (cases n. 8 and n. 9) and 5–10 ng/mL (case n. 10). A further 1/13 patients (case n. 11) had two LTC metastases occult at WBS with undetectable thyroglobulin levels. Moreover, the remaining 2/13 patients presented one LTC and one SM metastasis (case n. 12) and one LTC and one SC metastasis (case n. 13) at SPECT/CT, all of these occult at WBS, with thyroglobulin levels <2.5 and >10 ng/mL, respectively. Case n. 13 reported in Table 4 is illustrated in Figure 4 at radioiodine ablation and in Figure 5 at the follow-up.

## 4. Discussion

In the present, retrospective study on DTC patients already submitted to total thyroidectomy and radioiodine ablation, ^131^I-SPECT/CT performed 5–7 days after a therapeutic dose, proved a reliable procedure to better identify and characterize radioiodine-avid foci. The procedure also localized their anatomic site with better accuracy in respect to WBS, either thyroid tissue residues or neck LN metastases not removed during surgical dissection as well as distant metastases. Thus, SPECT/CT provided useful information on patient staging, risk stratification, prognosis and therapeutic strategy.

SPECT/CT after therapeutic radioiodine dose was also able to evidence a significantly more elevated number of radioiodine avid foci than WBS, many of which occult at the latter procedure. SPECT/CT also could clarify those foci classified as unclear or wrongly interpreted at planar scintigraphy correctly characterizing foci and thus reducing false positive and false negative cases. Namely, SPECT/CT was able to reclassify as malignant nodes some foci considered benign at WBS or vice versa to reclassify as benign some foci considered suspect of malignancy. Moreover, post-therapeutic SPECT/CT was capable of evidencing a significantly higher number of metastases in respect of WBS in 17 patients, most of whom with an age ≥ 45 years and tumor size >10 mm. Moreover, two of these cases were VL and six with TNM system of T1aN0M0 at surgery, and thyroglobulin levels were very low or undetectable in 5/17 patients at ablation after therapeutic dose, excluding one patient in whom undetectable thyroglobulin levels were associated to high AbTg.

Globally, SPECT/CT proved to increase the performance of WBS obtaining an incremental value and contributing to guiding the patients to a more appropriate therapeutic strategy improving their management during follow-up. In particular, SPECT/CT gave more information than WBS about risk stratification and TNM classification, which were correctly modified at post-therapeutic exams in respect to the results obtained during surgery modifying the number of neck LN or distant metastases in 64.7% of patients. These data are especially significant in VL and T1aN0M0 cases and even more when the foci are single and thyroglobulin levels are undetectable or very low, thus confirming that thyroglobulin values can be very low or undetectable also when a metastatic lesion is present, as previously reported in the literature [9,54,55].

The higher performance of SPECT/CT in respect of WBS has also been confirmed in those patients who could be monitored in the long-term follow-up, such as those 13/17 cases in whom SPECT/CT had identified metastases already in the exams performed after therapeutic radioiodine dose during ablation. To these latter patients, it was possible to add further 13 cases in whom only thyroid tissue residues had been ascertained at post-therapeutic scintigraphies, while metastases only appeared during follow-up. SPECT/CT in these two groups of patients was able, also after a radioiodine diagnostic dose, to give useful information to early identify any change of the disease status. In particular, in the former group, SPECT/CT was able to ascertain correctly disease progression or stable condition or also disease improvement with partial or total response to radioiodine treatment performed during the first ablation and always considering the useful support of the thyroglobulin values. At the same time, SPECT/CT could identify and characterize iodine-avid foci as local LN metastases during follow-up in the latter group of patients, foci that resulted unclear or occult at WBS. The appearance of these latter lesions, which were single in 10/13 cases, caused to change previous classification N0, performed at surgery and confirmed at therapeutic scintigraphy, to N1. In these patients, thyroglobulin levels not always increased with metastasis appearance, but even they remained very low or undetectable notwithstanding the presence of a metastatic neck LN, as evidenced in the 2 VL and T1aN0M0 cases in whom thyroglobulin levels were <2.5 ng/mL, thus confirming that not significant thyroglobulin values can be associated with WBS false-negative results. However, in some cases, low thyroglobulin levels associated with high values of AbTg could not exclude the presence of metastases. Thus, in the patients with positive AbTg observed in the present study, the role of SPECT/CT resulted even more important since this procedure, but not WBS, was able to evidence and/or characterize metastases clinically unsuspected and with undetectable thyroglobulin levels. 

Moreover, some new metastases also appeared after a long time (48 and 54 months later from diagnosis in the two aforementioned cases VL and T1aN0M0). This result would seem to confirm the slow growth of the thyroid carcinoma even if the probable presence of micrometastases below the spatial resolution of the gamma camera could not be excluded at the first therapeutic exam of SPECT/CT and WBS. Thus, a long-term follow-up is necessary to predict when an occult lesion may become clinically evident.

The favorable results of SPECT/CT and the incremental benefit over WBS obtained in this study agree with the data reported in previous studies in both pre [3,38,39] and post [40,41,42,43,44,45,56,57] ablation phases as well as during long-term follow-up [9,10,46,47,48,49,50], including in the latter cases data obtained with routine use of the procedure [9,10,47].

However, some limitations due to the retrospective nature of this study should be examined. Sub-centimetric lesions can cause difficulty in their identification because of the low spatial resolution of the gamma camera. The appearance of neck acquisition defects must be considered; however, these defects could be reduced by the valuable block system used for immobilization. Additional imaging time can cause discomfort to the patient. A more elevated exposition dose with associated use of CT can be present; however, the effective dose results low [58] and is outweighed by the benefits given from the procedure. Nuclear medicine experts belonging to only a Center have interpreted SPECT/CT images and therefore the results of the study cannot be generalizable. The patients have been studied in different conditions of thyroid function since these were under hypothyroidism after thyroid hormone interruption in the post-therapeutic scintigraphy. However, some patients during follow-up were under hypothyroidism while others after rhTSH stimulation; given the slow growth of DTC, a metastatic lesion can be evidenced in a late phase while it can appear negative at a first exam and positive later. This latter result happened in some patients of the present study and in particular in the two aforementioned cases, VL and T1aN0M0, in whom metastases appeared several months from initial diagnosis. Finally, the lack of histopathologic findings of some radioiodine-avid foci evidenced by SPECT/CT was realized owing to the difficulty of reaching the potential site of the lesions. The latter only could be validated by the data obtained in the follow-up with clinical exams, thyroglobulin sequential variations and radiologic and nuclear medicine imaging.

Despite these limitations, SPECT/CT with therapeutic dose during ablation phase proved a useful tool to identify and characterize metastatic lesions from DTC, both loco-regional and distant metastases, permitting a correct anatomic localization of the lesions and contributing to modify risk stratification and TNM of the patients with metastases in respect of the classification fixed at the surgery. In this way, SPECT/CT proved useful support for WBS image interpretation with an elevated impact on the management and therapeutic strategy of affected patients. Moreover, SPECT/CT also demonstrated high performance during long-term follow-up even with radioiodine diagnostic dose identifying metastatic neck LN, single in most cases and modifying patient staging and therapeutic strategy whereas WBS role has been inconclusive.

SPECT/CT proved a simple and non-invasive procedure always giving high-quality images and enough easy to be interpreted with adequate training. 

In the present study, some risk factors identified at the surgery have been considered to evaluate their involvement in the appearance of metastases ascertained both at SPECT/CT after the therapeutic dose at ablation and after diagnostic dose during follow-up. In particular, the presence of neck LN metastases and ETE at surgery were evaluated, the latter risk factor including eETE and mETE. Associating the results of SPECT/CT at different time points, post-therapeutic and follow-up phases, and excluding 16/106 patients with only residues at post-therapeutic scintigraphies and lost in the follow-up, metastases have been identified in 30/90 (33.3%) of cases, 10 patients with primary tumor size ≤ 10 mm and 20 with tumor size > 10 mm. Of these patients, 11/30 (36.7%) had at surgery the risk factor ETE (one with eETE and 10 with mETE) and further 7/30 (23.3%) cases had neck LN (N1b) metastasis risk. Considering the ETE risk factor, the only patient with eETE and with primary tumor > 15 mm in size, presented a marked worsening of prognosis with the appearance of local and distant metastases and died for thyroid cancer confirming the negative prognostic impact of eETE risk factor on metastases also described in other studies on wide casuistries [13,14,15,16]. Concerning the 10/30 (33.3%) patients who had mETE at surgery, it resulted that also this risk factor is associated with a poor prognosis (*p* = 0.027) and at the univariate analysis the affected patients showed an increased risk for metastasis appearance after thyroidectomy, OR 3.25 (95% CI 1.13–9.41), *p*-value = 0.03. These results referring to the few cases of the present study confirm previous data reported in the literature [9,17,18,19,20,21] while differing from others [13,22,23,24,25,26,27,28]. Moreover, the primary tumor size of mETE patients was >15 mm in 9/10 (90%) of cases and <10 mm in the remaining case, the latter being a microcarcinoma (8 mm). These data confirm, for most of the cases, the previous results reported by some authors [27,59] for whom mETE represents an unfavorable prognostic factor only in larger tumors even if in the present casuistry there was one exception represented by a microcarcinoma.

Considering the risk factor neck LN metastasis already present at the surgery in 7 patients, these developed further metastases in the follow-up (*p* = 0.029); at univariate analysis, they presented an increased risk for metastases, OR 4.26 (95% CI 1.14–15.96), *p*-value = 0.031.

Thus, we can hypothesize that the patients affected by thyroid carcinomas with these two aforementioned risk factors can potentially develop metastases during follow-up after surgery. Moreover, 10/30 (33.3%) of patients with metastases also had a multifocal carcinoma, as well as further 6/30 (20%) of the cases, were also affected by GB or HT hypothesizing that even these abnormalities might contribute to the progression of tumor disease, as described in previous reports [9,60]. At the same time, also the size of the primary tumor might present an effect on metastasis appearance during follow-up since 66.7% of metastatic patients observed in the present study had a carcinoma > 10 mm, as also reported in previous data of literature [10,18,20].

Based on the results of the present study, DTC patients who should be carefully monitored during follow-up are those who presented not only eETE but also mETE and neck LN metastases and even more when multifocal carcinoma, GB and HT were also associated at the surgery as well as tumor diameter >10 mm. 

## 5. Conclusions

^131^I-SPECT/CT in the present study proved higher performance than WBS in both post-radioiodine and follow-up phases of DTC patients to establish correct staging and risk stratification, assess residues and evaluate disease progression and regression. In particular, SPECT/CT was also able to identify new neck lymph node metastases, occult (87.5%) or unclear (12.5%) at WBS, in patients in long-term follow up who had been classified as N0 at surgery and at the first radioiodine ablation. Among the latter, some cases with microcarcinoma and very low levels of serum thyroglobulin, and absence of risk factors were included suggesting that is necessary not to underestimate also this type of tumor during follow up. Therefore, routine SPECT/CT use is suggested as a complementary role to the planar method in the diagnostic protocol of DTC since, in the present casuistry, the metastases were often unclear or occult at WBS and were characterized only by SPECT/CT. Moreover, careful surveillance of affected patients should be guaranteed in a long-term follow-up since new metastases can appear many months after surgery and radioiodine ablation. In particular, it is necessary to take an even greater cure when some tissue risk factors are present at surgery, such as extrathyroid tumor extension, also minimal and neck lymph node metastases, which seem to represent unfavorable prognostic factors.

## Figures and Tables

**Table 1:** Clinical and demographic characteristics of 106 patients with differentiated thyroid carcinoma (DTC) at the surgery.

Characteristics	Patients (*n*)	Characteristics	Patients (*n*)
Sex		Structural characteristics	
Male	29	Unifocal carcinoma	62
Female	77	Multifocal carcinoma	44
Age		Minimal tumor extra-thyroid extension	21
<45 y	33	Extended tumor extra-thyroid extension	1
≥45 y	73	Lymph node metastasis	14
Histology		Distant metastasis	2
Papillary carcinoma	98	Chronic thyroiditis	20
Follicular carcinoma	3	Graves/Basedow disease	5
Hürtle cell carcinoma	5	Risk stratification	
Size		High risk	29
≤10 mm	42	Low risk	56
>10 mm	64	Very low risk	21

**Table 2:** Radioiodine-avid foci evidenced and characterized by SPECT/CT (n. 260) and by WBS (n. 231) during ablation.

Foci Concordantly Classified by WBS and SPECT/CT	Foci Unclear at WBS Characterized by SPECT/CT	Foci Occult (oc) at WBS Characterized by SPECT/CT	Foci Wrongly (W) Classified by WBS Correctly Characterized by SPECT/CT
n. 186	n. 42	n. 29	n. 3
172 Residues 8 Lung metastases 5 Soft tissue metastases 1 Cutaneous contamination	24 Residues 10 Neck lymph node metastases 6 Lymph node metastases outside the neck 1 Bone metastases 1 Physiologic uptake (stomach)	12 Residues 12 Neck lymph node metastases 1 LN metastasis outside the neck 4 Lung metastases	1 Neck lymph node metastasis 1 Thorax muscle metastasis 1 Benign vertebral disorder

**Table 3:** A total number of metastases evidenced by SPECT/CT (n. 49) in 17/106 patients and by WBS (n. 32) in 13/17 patients at radioiodine ablation; 13/17 cases (cases 1–13) also monitored during follow-up, underwent progression (cases 1–4), stabilization (cases 5–8) and improvement (cases 9–13) of the disease.

AT SURGERY												AT RADIOIODINE ABLATION					AT FOLLOW-UP				
Patients n.	Sex	Age	Histology	Size (mm)	Focality	ETE	Metastases	HT	GB	TNM	Risk Stratification	Thyroglobulin (ng/mL)	Planar WBS (n. foci)	SPECT/CT (n. foci)	TNM	Risk Stratification	Thyroglobulin (ng/mL)	Planar WBS (n. foci)	SPECT/CT (n. foci)	TNM	Risk Stratification
1	F	<45	PC	≤10	UF			HT		T1aN0M0	VL	und + AbTg	1 unclear	2 LTC (1 oc)	T1aN1M0	H	und + AbTg	0	3 LTC (oc)	T1aN1M0	H
2	M	<45	PC	>10	UF	mETE				T3N0M0	H	>10.0	2 unclear	2 LTC	T3N1M0	H	>10.0	0	3 LTC (oc)	T3N1M0	H
3	F	≥45	PC	≤10	MF					T1aN0M0	L	und	1 unclear	1 M	T1aN0M1	H	>5.0–10.0	1 unclear	1 M, 1 LTC (oc)	T1aN1M1	H
4	F	<45	PC	>10	UF	mETE	LN			T3N1M0	H	>10.0	2 unclear	2 LTC	T3N1M0	H	>10.0	2 unclear	2 LTC, 1 SM (oc), 1 lung (oc), 1 bone (oc)	T3N1M1	H
5	F	≥45	PC	≤10	MF					T1aN0M0	L	2.5–5.0	1 unclear	1 PT	T1aN1M0	H	2.5–5.0	0	1 PT (oc)	T1aN1M0	H
6	F	<45	PC	>10	MF		LN			T1bN1M0	H	>10.0	0	1 PT (oc)	T1bN1M0	H	>5.0–10.0	0	1 PT (oc)	T1bN1M0	H
7	M	≥45	PC	>10	UF	eETE	LN + DM			T4N1M1	H	>10.0	6 unclear, 1 lung, 5 ST	1 LTC, 1 bone, 5 AP (1 oc), 1 lung, 5 ST	T4N1M1	H	>10.0	6 unclear, 1 lung, 5 ST	1 LTC, 1 bone, 5 AP (1 oc), 1 lung, 5 ST	T4N1M1	H
8	M	≥45	PCFV	>10	UF	mETE	LN			T3N1M0	H	>10.0	1 lung	1 lung, 1 PT (oc)	T3N1M1	H	>10.0	1 lung	1 lung, 1 PT (oc)	T3N1M1	H
9	F	<45	PC	>10	MF			HT		T1bN0M0	L	>10.0	0	3 LTC (oc)	T1bN1M0	H	>5.0–10.0	0	2 LTC (oc)	T1bN1M0	H
10	F	<45	PC	>10	UF	mETE	LN	HT		T3N1M0	H	>10.0	1 unclear	2 LTC (1 oc)	T3N1M0	H	<2.5	0	1 LTC (oc)	T3N1M0	H
11	F	≥45	PC	≤10	MF			HT		T1aN0M0	L	<2.5	0	3 LTC (oc), 1 PT (oc)	T1aN1M0	H	und	0	1 PT (oc)	T1aN1M0	H
12	M	≥45	PCFV	>10	UF		DM		GB	T4N0M1	H	>10.0	5 lung, 1 W	5 lung, 1 TM (W)	T4N0M1	H	>5.0–10.0	1 lung	1 lung	T4N0M1	H
13	F	≥45	PC	≤10	UF					T1aN0M0	VL	>10.0	1 unclear	1 SM	T1aN1M0	H	und	0	0	T1aN0M0	VL
14	F	<45	PC	≤10	MF					T1aN0M0	L	<2.5	0	1 lung (oc)	T1aN0M1	H					
15	M	≥45	PC	>10	UF	mETE	LN			T3N1M0	H	>10.0	1 lung, 1 unclear	4 lung (3 oc), 1 M	T3N0M1	H					
16	F	≥45	PC	>10	UF					T1bN0M0	L	und	1 unclear	1 SC	T1bN1M0	H					
17	M	≥45	PC	>10	MF	mETE	LN			T3N1M0	H	<2.5	1 W	1 SM (W), 1 LTC (oc)	T3N1M0	H					

PC: papillary carcinoma; LN: lymph node metastasis; und: undetectable thyroglobulin; PT: paratracheal lymph node metastasis; PCFV: papillary carcinoma follicular variant; DM: distant metastasis; AbTg: anti-thyroglobulin antibodies; SM: sub-mandibular lymph node metastasis; UF: unifocality; HT: Hashimoto thyroiditis; lung: lung metastasis; SC: supra-clavicular lymph node metastasis; MF: multifocality; GB: Graves-Basedow disease; bone: bone metastasis; M: mediastinum lymph node metastasis; ETE: tumor extra-thyroid extension; H: high risk; TM: thorax muscle metastasis; AP: abdomen/pelvis lymph node metastasis; mETE: minimal tumor extra-thyroid extension; L: low risk; ST: soft-tissue metastasis; oc: occult at WBS; eETE: extended tumor extra-thyroid extension; VL: very low risk; LTC: laterocervical lymph node metastasis; W: wrongly classified at WBS.

**Table 4:** ^131^I-SPECT/CT and WBS and thyroglobulin levels assay in 13 DTC patients with absence of metastases both at surgery and at radioiodine ablation imaging, undergone neck lymph node metastases during follow-up.

AT SURGERY										AT RADIOIODINE			AT FOLLOW-UP				
Patient s n.	Sex	Age	Histology	Size (mm)	Focality	ETE	GB	TNM	Risk Stratification	Thyroglobulin (ng/mL)	Planar WBS (n. foci)	SPECT/CT (n. foci)	Thyroglobulin (ng/mL)	Planar WBS (n. foci)	SPECT/CT (n. foci)	TNM	Risk Stratification
1	F	<45	PC	≤10	UF			T1aN0M0	VL	und	2 R	2 R	<2.5	1 unclear	1 SM	T1aN1M0	H
2	F	≥45	PC	≤10	UF			T1aN0M0	VL	und	1 R	1 R	<2.5	0	1 SM (oc)	T1aN1M0	H
3	F	<45	PC	≤10	UF			T1aN0M0	VL	und + AbTg	1 R	1 R	und + AbTg	0	1 SM (oc)	T1aN1M0	H
4	F	<45	PC	≤10	UF	mETE	GB	T3N0M0	H	2.5–5.0	2 R	2 R	2.5–5.0	0	1 SM (oc)	T3N1M0	H
5	F	≥45	PC	>10	UF	mETE		T3N0M0	H	2.5–5.0	1 R	1 R	2.5–5.0	0	1 SM (oc)	T3N1M0	H
6	M	≥45	PC	>10	MF	mETE		T3N0M0	H	2.5–5.0	1 R	1 R	2.5–5.0	0	1 SM (oc)	T3N1M0	H
7	F	<45	PC	>10	UF			T1bN0M0	L	und	1 R	1 R	<2.5	0	1 LTC (oc)	T1bN1M0	H
8	F	≥45	PC	>10	UF			T1bN0M0	L	und + AbTg	2 R	2 R	und + AbTg	1 unclear	1 LTC	T1bN1M0	H
9	F	<45	PC	>10	UF			T1bN0M0	L	und + AbTg	3 unclear	3 R	und + AbTg	0	1 LTC (oc)	T1bN1M0	H
10	F	<45	PC	>10	UF			T1bN0M0	L	<2.5	4 R	4 R	>5.0–10.0	1 unclear	1 LTC	T1bN1M0	H
11	F	≥45	PC	>10	UF	mETE		T3N0M0	H	und	3 R	4 R	und	0	2 LTC (oc)	T3N1M0	H
12	F	≥45	PC	>10	MF			T1bN0M0	L	und	1 R	2 R	<2.5	0	1 LTC (oc), 1 SM (oc)	T1bN1M0	H
13	F	≥45	PC	>10	MF			T1bN0M0	L	2.5–5.0	2 R	2 R	>10.0	0	1 LTC (oc), 1 SC (oc)	T1bN1M0	H

PC: papillary carcinoma; ETE: tumor extra-thyroid extension; H: high risk; R: residue; UF: unifocality; mETE: minimal tumor extra-thyroid extension; L: low risk; SM: sub-mandibular lymph node metastasis; MF: multifocality; HT: Hashimoto thyroiditis; VL: very low risk; LTC: laterocervical lymph node metastasis; GB: Graves-Basedow disease; und: undetectable thyroglobulin; SC: supra-clavicular lymph node metastasis; AbTg: anti-thyroglobulin antibodies; oc: occult at WBS.

## Data Availability

The data presented in this study are available on reasonable request from the corresponding author.
